# Microalgae as Sustainable Bio-Factories of Healthy Lipids: Evaluating Fatty Acid Content and Antioxidant Activity

**DOI:** 10.3390/md19070357

**Published:** 2021-06-23

**Authors:** Tiago A. Conde, Bruna F. Neves, Daniela Couto, Tânia Melo, Bruno Neves, Margarida Costa, Joana Silva, Pedro Domingues, M. Rosário Domingues

**Affiliations:** 1 Mass Spectrometry Centre, LAQV-REQUIMTE, Department of Chemistry, University of Aveiro, Santiago University Campus, 3810-193 Aveiro, Portugal; tiagoalexandreconde@ua.pt (T.A.C.); brunabneves@ua.pt (B.F.N.); danielacouto@ua.pt (D.C.); taniamelo@ua.pt (T.M.); p.domingues@ua.pt (P.D.); 2 CESAM—Centre for Environmental and Marine Studies, Department of Chemistry, University of Aveiro, Santiago University Campus, 3810-193 Aveiro, Portugal; 3 Department of Medical Sciences and Institute of Biomedicine—iBiMED, University of Aveiro, 3810-193 Aveiro, Portugal; bruno.neves@ua.pt; 4 Allmicroalgae Natural Products S.A., R&D Department, Rua 25 de Abril 19, 2445-287 Pataias, Portugal; costa.anamarg@gmail.com (M.C.); joana.g.silva@allmicroalgae.com (J.S.)

**Keywords:** microalgae, lipidomics, lipids, fatty acids, mass spectrometry, GC-MS

## Abstract

The demand for sustainable and environmentally friendly food sources and food ingredients is increasing, and microalgae are promoted as a sustainable source of essential and bioactive lipids, with high levels of omega-3 fatty acids (ω-3 FA), comparable to those of fish. However, most FA screening studies on algae are scattered or use different methodologies, preventing a true comparison of its content between microalgae. In this work, we used gas-chromatography mass-spectrometry (GC-MS) to characterize the FA profile of seven different commercial microalgae with biotechnological applications (*Chlorella vulgaris*, *Chlorococcum amblystomatis*, *Scenedesmus obliquus*, *Tetraselmis chui*, *Phaeodactylum tricornutum*, *Spirulina* sp., and *Nannochloropsis oceanica*). Screening for antioxidant activity was also performed to understand the relationship between FA profile and bioactivity. Microalgae exhibited specific FA profiles with a different composition, namely in the ω-3 FA profile, but with species of the same phylum showing similar tendencies. The different lipid extracts showed similar antioxidant activities, but with a low activity of the extracts of *Nannochloropsis oceanica*. Overall, this study provides a direct comparison of FA profiles between microalgae species, supporting the role of these species as alternative, sustainable, and healthy sources of essential lipids.

## 1. Introduction

Microalgae are considered a sustainable source of essential and bioactive lipids [1]. Moreover, they are quite diverse in their fatty acid (FA) profile and abundances, which shows exploitable potential [2]. In fact, different saturated FA (SFA), monounsaturated FA (MUFA), and polyunsaturated FA (PUFA) were described in microalgae. However, there are some common FAs observed among different microalgae, for example, hexadecanoic acid (C16:0) and oleic acid (C18:1). Some microalgae are rich in omega-3 (ω-3) FA), and are often considered as the main producers of these healthy lipids, representing an alternative source to fish [3]. Depending on the microalgae, they can provide essential FAs, such as alpha-linolenic acid (C18:3 ω-3; ALA), which can be metabolized in mammals to eicosapentaenoic acid (C20:5 ω-3; EPA) and docosahexaenoic acid (C22:6 ω-3; DHA) [4]. Some microalgae also provide EPA directly and are therefore a direct source of this healthy and bioactive FA [5]. This is of the utmost importance as EPA is not found at high levels in terrestrial plants but is found in some seaweeds, such as *Palmaria palmata* [6], but in lower amounts compared to microalgae [5].

Omega-3 FAs are important nutrients for the central nervous system in humans and other mammals [7,8]. They are also important antioxidant compounds and are recognized precursors of anti-inflammatory eicosanoids [9], thus playing a key role in the prevention of neurodegenerative diseases and memory loss [10,11] and in the regulation of inflammation [9]. Their ability to participate in the modulation of inflammation is quite important in the prevention of non-communicable diseases (NCDs) [12], such as cardiovascular disease, diabetes, and obesity. These NCDs are among the main causes of morbidity and mortality in the world, being associated with chronic low-grade inflammation characterized by continuously elevated levels of circulating pro-inflammatory cytokines, chemokines and acute inflammatory phase proteins [13], and continuous oxidative stress [14], leading to impaired bodily function. In this context, the use of ω-3 FA to prevent and mitigate chronic inflammation offers an opportunity to reduce the prevalence of NCDs.

Modern diets often have an imbalance in the intake of these essential and healthy FA [15]. Microalgae are considered important new foods for the prevention of the global burden of 21st century chronic diseases, such as NCDs [16,17], and therefore should be included in healthy and sustainable diets [18,19]. The recommendation for microalgae intake is also supported by their sustainable production in aquaculture [20], as it does not compete with water or land and, consequently, does not compete with other terrestrial plants, reducing ocean pollution and overexploitation of marine resources [21]. The disadvantages of consuming fish oil, for example, the significant abundance of heavy metals or antibiotics in fish oils, also bolster microalgae as a promising source of healthy lipids [22]. Microalgae have the advantage of being single-cell factories, producing sustainable and safe biomass and ingredients with reproducible nutritional and health value for food, ingredients or supplements, feed or pharmaceuticals, and cosmetics [3].

Nowadays, most of the microalgae used in the food industry are *Chlorella vulgaris, Odontella aurita,* and *Spirulina*, typically as food supplements, and *Tetraselmis chui*, as a flavoring agent for seafood [23]. Other microalgae are used to obtain extracts for a wide range of uses (such as supplementation with essential nutrients, pharmaceutical and cosmetic applications, production of biodiesel, among others), thus they have been studied for many years [24,25,26]. The presence of high abundances of ω-3 FA in microalgae is essential to valorize them as food products and food ingredients [27]. Omega-3 FA are sensitive to oxidation and could, therefore, work as food preservatives to prevent spoilage [28]. Moreover, microalgae are recognized as a source of antioxidant compounds, namely, polyphenols [29]. However, despite the antioxidant potential of lipids in microalgae, they remain scarcely recognized, and most studies explore the use of aqueous extracts of microalgae, rich in phenolic compounds [29,30]. Nevertheless, individual studies on microalgae have highlighted and recognized the antioxidant potential of their healthy lipids [31,32,33], for example, FA from different microalgae from different phyla.

Over the years, numerous studies have reported that microalgae have different FA profiles, some of them considered important for algal exploitation and nutritional valorization as well as for taxonomic classifications [34]. Indeed, it has already been reported that the level of production of ω-3 FA and FA species differs between microalgae. For example, the taxa of *Diatoms* and *Eustigmatophytes*, such as *Phaeodcatylum tricornutum* and *Nannochloropsis* sp., respectively, are important synthesizers of EPA while microalgae belonging to the taxa of *Dinophytes, Haptophytes*, and *Thraustochytrids* are considered to be producers of DHA- [35,36,37]. The production of long-chain ω-3 FA (EPA and DHA) in *Cyanobacteria* (e.g., *Spirulina platensis*) and *Chlorophytes* (e.g., *Chlorella vulgaris*) is considered negligible and, in the case of *C. vulgaris*, only interesting amounts of ALA or stearidonic acid (C18:4 ω-3) are found [38]. Although some microalgae produce lower levels of ω-3 FA, these amounts can be increased by manipulating growing conditions [39], since the production of ω-3 FA is also dependent on the surrounding environment [40]. Therefore, the characterization of the FA profile from microalgae is of the utmost importance.

Nevertheless, published work focused on the study of microalgae FA is scattered because different authors have used different extraction methods, FA derivatization techniques, and data acquisition [31,32,41,42,43]. For example, published work has often analyzed crude extracts, obtained using different mixtures of solvents with different extraction capacities, or different extraction methodologies. In fact, the vast majority of studies have used chloroform:methanol extractions and differences seem to stem from other ancillary procedures of mechanical cell disruption (e.g., ultrasound-assisted extraction, supercritical CO_2_ extraction) that are necessary with some species of hard cell walls and to enhance the extraction of lower yield solvents (e.g., ethanol) [44]. Additionally, most studies used a gas-chromatography flame ionization detector (GC-FID); however, GC-mass spectrometry (MS) is considered a more powerful and accurate methodology currently available to detect FA (e.g., gas-chromatography mass spectrometry (GC-MS)) [45].

Thus, in this work, an overview of the microalgae most commonly used as biomass or ingredients will be given, using an efficient lipid extraction method, aimed at clarifying the FA profiles according to the species of microalgae, grown under saline (*Nannochloropsis oceanica*, *Phaeodactylum tricornutum*, *Tetraselmis chui*) or fresh water media (*Chlorella vulgaris*, *Chlorococcum amblystomatis*, *Scenedesmus obliquus*, *Spirulina* sp.), but also looking for putative correlation between the fatty acid profile and antioxidant activity of the extracts. The selected microalgae are among the most cultivated species in Europe [46]. The present work will thus promote the valorization of microalgae as food and feed ingredients with added value or products of pharmacological interest.

## 2. Results

### 2.1. Lipid Content in Different Microalgae

**Figure 1:**
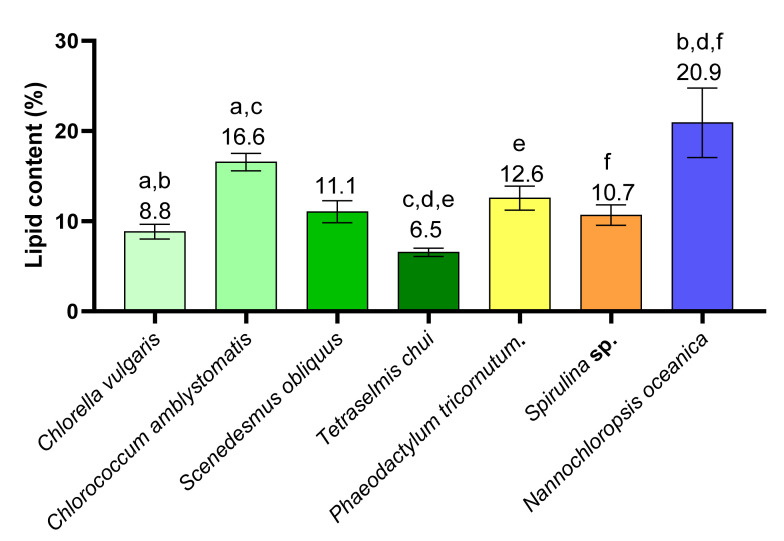
Lipid content of *Chlorella vulgaris*, *Chlorococcum amblystomatis*, *Scenedesmus obliquus*, *Tetraselmis chui*, *Phaeodactylum tricornutum*, *Spirulina* sp., and *Nannochloropsis oceanica* (expressed in percentage % of biomass). Matching letters (a–f) indicate statistically significant differences between microalgae species, i.e., the same letter represents significant differences (*q* < 0.05, Kruskal–Wallis test followed by Dunn’s post-hoc comparisons).

The lipid content of the microalgae *Chlorella vulgaris*, *Chlorococcum amblystomatis*, *Scenedesmus obliquus*, *Tetraselmis chui*, *Phaeodactylum tricornutum*, *Spirulina* sp., and *Nannochloropsis oceanica* were determined by gravimetry and are described in Figure 1 and Table S1. *N. oceanica* was the microalgae with the highest lipid content (20.9 ± 3.4%) followed by *C. amblystomatis*, *P. tricornutum*, *S. obliquus*, and *Spirulina* sp., respectively. The microalgae *C. vulgaris* and *T. chui* had the lowest lipid contents (8.8 ± 0.7% and 6.5 ± 0.4%, respectively). The lipid content of *N. oceanica* was significantly different from that of C. vulgaris, *T. chui*, and *Spirulina* sp. (*q* < 0.05). In addition, the lipid content of *C. vulgaris* was significantly lower than that of *C. amblystomatis*, and the latter was significantly higher than that of *T. chui*. The microalgae *T. chui* had a significantly lower lipid content than that of *P. tricornutum*.

### 2.2. Fatty Acid Profile of Different Microalgae and Nutritional Indices

The FA profiles identified in the total lipid extracts of *C. vulgaris*, *C amblystomatis*, *S. obliquus*, *T. chui*, *P. tricornutum*, *Spirulina* sp., and *N. oceanica* are summarized in Table 1. The FAs were identified after transesterification reactions and analyzed as fatty acid methyl esters (FAMEs) using gas-chromatography mass spectrometry (GC-MS). One of the most abundant FAs identified in all microalgae was palmitic acid (C16:0), with a higher abundance in *Spirulina* sp. extracts (38.6 ± 0.4%). Other FAs were common among the selected microalgae, such as C14:0, C16:1 ∆^9^ (ω-7), C17:0, C18:0, C18:1 ∆^11^ (ω-7), C18:1 ∆^9^ (ω-9), and C18:2 ∆^9,12^ (ω-6). Some of the common FAs displayed a higher relative abundance in different species, namely C16:1 ∆^9^ (ω-7) in *P. tricornutum* (16.4 ± 0.3%) and *N. oceanica* (21.6 ± 1.3%), and both C18:2 ∆^9,12^ (ω-6) and C18:3 ∆^6,9,12^ (ω-6) in *Spirulina* sp. (21.4 ± 0.8% and 23.3 ± 0.8%, respectively). In addition to these FAs, C16:4 ∆^4,7,10,13^ (ω-3) was identified with a high abundance in *C. amblystomatis*, *S. obliquus*, and *T. chui* (12.7 ± 0.6%, 15.5 ± 1.4%, and 14.5 ± 1.8%).

With the exception of *Spirulina* sp., all microalgae had different but interesting amounts of distinct ω-3 FA, namely C18:4 ∆^6,9,12,15^ (ω-3), α-linolenic acid (C18:3 ∆^9,12,15^ (ω-3), ALA) and eicosapentaenoic acid (C20:5 ∆^5,8,11,14,17^ (ω-3), EPA). The lipid extract of S. obliquus had the highest relative abundance of ALA (35.0 ± 2.7%) followed by *C. vulgaris* (26.3 ± 1.4%), *C. amblystomatis* (21.9 ± 0.9%), *T.chui* (18.3 ± 2.4%), and *P. tricornutum* (1.8 ± 0.1%), which had the lowest abundance. EPA was identified in four microalgae, with *N. oceanica* having the highest relative abundance (30.8 ± 2.4%) followed by *P. tricornutum* (27.3 ± 1.5%), *C. amblystomatis* (10.7 ± 0.6%), and *T.chui* (4.2 ± 0.6%). Among the selected microalgae, only *P. tricornutum* had docosahexaenoic acid (C22:6 ∆^4,7,10,13,16,19^ (ω-3), DHA), with a relative abundance of 0.6 ± 0.1%. 

**Figure 2:**
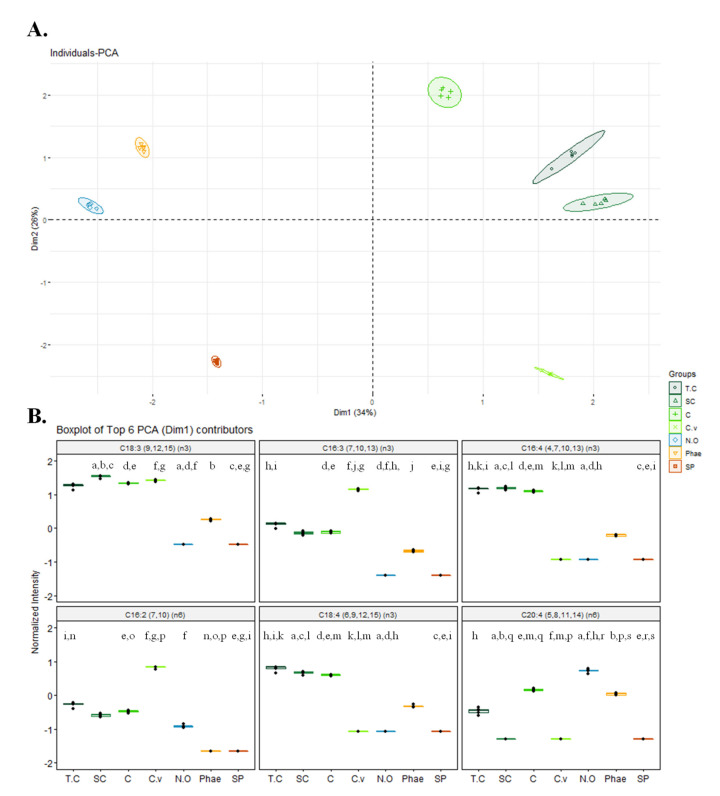
(**A**) Principal component analysis (PCA) scores plot and (**B**) Boxplots of the 6 major contributors for PC1 using the relative abundance after log normalization of all fatty acids identified in *Chlorella vulgaris*, *Chlorococcum amblystomatis*, *Scenedesmus obliquus*, *Tetraselmis chui*, *Phaeodactylum tricornutum*, *Spirulina* sp., and *Nannochloropsis oceanica*. Abbreviations: C, *Chlorococcum amblystomatis*; C.v, *Chlorella vulgaris*; SC, *Scenedesmus obliquus*; T.C, *Tetraselmis chui*; Phae, *Phaeodactylum tricornutum*; SP, *Spirulina* sp.; and N.O, *Nannochloropsis oceanica*. Matching letters (a–s) indicate statistically significant differences between microalgae species, i.e., the same letter represents significant differences (*q* < 0.05, Kruskal–Wallis test followed by Dunn’s post-hoc comparisons).

The relative abundance of all FAs detected in the seven microalgae was used for multivariate statistical analysis. The hierarchical clustering and principal component analysis (PCA) plots are shown in Figure 2 and Figure S1, respectively. Hierarchical clustering showed that the microalgae of the Chlorophytes phylum clustered together at the first level of the tree and the other phylum (Ochophytes, Bacillariophytes, and Cyanobaceria) were merged. At the second level of the tree, *T. chui* and *S. obliquus*, and *C. amblystomatis* clustered together and *C. vulgaris* formed its own meta-class. With levels three and four, the other microalgae of the Chlorophytes formed their own meta-class. The other phyla were clustered into different branches, with Cyanobaceria (*Spirulina* sp.) forming its own meta-class at level two and Ochophytes (*N. oceanica*) and Bacillariophytes (*P. tricornutum*) forming their own meta-class at level three of the tree.

PCA analysis showed that the first two principal components accounted for 60% of the total variance (PC1 34%; PC2 26%). PCA results also showed an association of four microalgae belonging to the same phylum Chlorophyta (*C. vulgaris*, *C. amblystomatis*, *S. obliquus*, *T. chui*) along PC1, as shown in Figure 2. Among the six major contributors to discrimination, four were omega-3 PUFA: C18:3 ω-3, C16:3 ω-3, C16:4 ω-3, and C18:4 ω-3. The relative abundance of these FAs was higher in species of green microalgae but specific for each microalga, namely C18:3 ω-3 in *C. vulgaris*, *C. amblystomatis*, *S. obliquus,* and *T. chui*; C16:3 ω-3 in *C. vulgaris*; and C16:4 and C18:4 ω-3 in *C. amblystomatis*, *S. obliquus*, and *T. chui*.

The Thrombogenicity Index (TI), Atherogenicity Index (AI), PUFA ω-6/ω-3, and hypocholesterolemic/hypercholesterolemic (h/H) ratios were calculated from FA data obtained to assess the nutritional index and the potential health benefits of these seven microalgae (Table 2). The values of the AI and TI indices varied between 0.2 and 0.7, and 0.1 and 1.6, respectively. *S. obliquus* (0.2 ± 0.1) and *C. vulgaris* (0.2 ± 0.0) recorded the lowest AI, with significant differences detected between these values and those of microalgae belonging to different phylum (Bacillariophyta, Cyanobacteria, and Ochrophyta). The lowest TI value also belonged to S. obliquus extracts (0.1 ± 0.0), being significantly different from *N. oceanica* and *Spirulina* sp. Regarding the h/H ratio, the highest value was obtained for *S. obliquus* followed by *C. vulgaris* and *P. tricornutum*. There are significant differences between *S. obliquus* and the other two microalgae, *Spirulina* sp. and *T. chui*. The microalgae *Spirulina* sp. had the lowest h/H value, being significantly different from *C. vulgaris*, *P. tricornutum*, and *S. obliquus*.

### 2.3. In Chemico Evaluation of Antioxidant Activity

**Figure 3:**
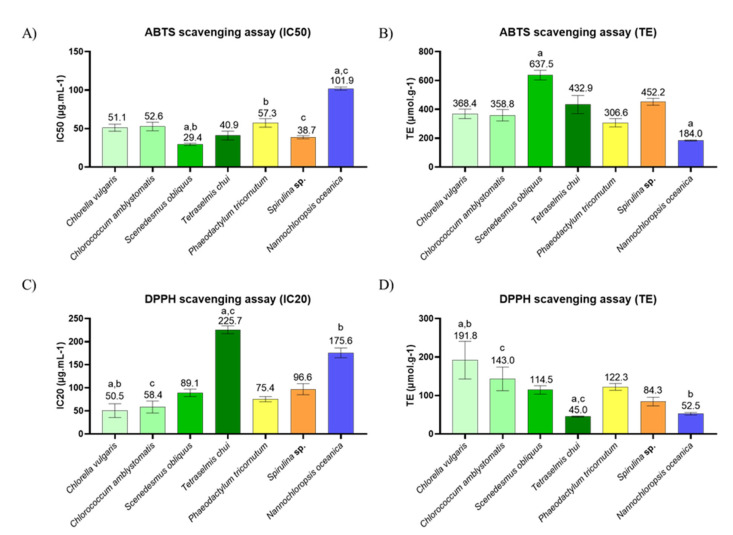
Evaluation of the antioxidant activity of lipid extracts from different microalgae. Concentration of lipid extract (µg.mL^−1^) that provided: (**A**) 50% inhibition of the ABTS^●+^ radical, (**B**) 20% inhibition of the DPPH^●^ radical, and (**C**,**D**) the Trolox equivalents (TE; µmol.g^−1^). The values are displayed as the mean (n = 3) ± standard deviation. Matching letters (a–c) represent significant differences between microalgae, i.e., the same letter represents significant differences (*q* < 0.05, Kruskal–Wallis test followed by Dunn’s post-hoc comparisons).

The antioxidant activity of the lipid extracts of *C. vulgaris*, *C. amblystomatis*, *S. obliquus*, *T. chui*, *P. tricornutum*, *Spirulina* sp., and *N. oceanica* was evaluated using the ABTS^●+^ and DPPH^●^ scavenging assays of the free radicals, and the results are shown in Figure 3 and Figure S2.

The lipid extracts of all the microalgae studied had an inhibition of 50% (IC50) in the ABTS^●+^ assay, at the tested concentrations. The lowest IC50 was obtained with *S. obliquus* (29.4 ± 1.2 μg/mL) and Trolox equivalents (TE) of 637.5 ± 27.4, followed by *Spirulina* sp., *T. chui*, *C. vulgaris*, *C. amblystomatis*, and *P.tricornutum*. *N. oceanica* extracts showed the highest IC50 (101.9 ± 1.7 µg.mL^−1^) and the lowest TE values (184.0 ± 3.2 µmol.g^−1^), compared to other extracts.

In the DPPH^●^ assay, all the microalgae studied were found to inhibit up to 20% (IC20) of the radical. The *C. vulgaris* extracts were responsible for the lowest IC20 value (50.5 ± 12.3, with a TE of 191.8 ± 40.0). The two extracts of *T. chui* and *N. oceanica* showed the highest IC20 (225.7 ± 6.9 µg.mL^−1^ and 175.6 ± 8.7 µg.mL^−1^, respectively) and the lowest TE (45.0 ± 1.4 µmol.g^−1^ and 52.5 ± 2.7 µmol.g^−1^, respectively) of microalgae extract.

## 3. Discussion

Microalgae are innovative food products with high nutritional value and are a rich source of essential nutrients, such as proteins, vitamins, minerals, carbohydrates, or lipids, contributing to a healthy and sustainable diet [19]. They are primary producers of ω-3 FA, thus representing a sustainable alternative to fish sources, and have current food applications [3]. Modern diets are known to be rich in saturated lipids and refined sugars but lacking in essential and healthy lipids, such as ω-3 FA [15]. These unbalanced diets cause malnutrition and increase the risk factor for noncommunicable diseases. Significant efforts are being made to encourage consumers to adopt healthier diets and new food products that provide essential, safe, and bioactive nutrients, such as algae.

The seven microalgae selected for this study belong to four different phylum, Chlorophyta (*C. vulgaris*, *C. amblystomatis*, *S. obliquus*, and *T.chui*), Bacillariophyta (*P. tricornutum*), Cyanobacteria (*Spirulina* sp.), and Ochrophyta (*N. oceanica*). Three of them (*C. vulgaris*, *T.chui*, and *Spirulina* sp.) have food safety approval for human consumption [23]. The microalga with the highest lipid content was *N. oceanica* (20.9 ± 3.4%), and the one with the lowest amount was *T. chui* (6.5 ± 0.4%), highlighting the great differences existing among the species. Microalgae lipid content is typically described as within 20–50% of the microalgae dry weight, which was not observed in the present work [47]. Probably, the difference in results could be due to the different growth conditions of the microalgae or due to the use of crude organic extracts, thus other molecules, other than lipids, could have contributed to the weight of the extract [48,49]. 

The FA described in this work correspond to esterified FAs, which are considered to be more bioaccessible when compared to their free forms [50]. The FAs profiled for these different microalgae were consistent with what has been reported in previous studies for each alga, concerning FA, but with differences in the FA contents [31,51,52,53,54,55,56], namely the present work reports higher amounts of ω-3 FA. These differences may be due to the different growth conditions for the microalgae studied, the use of different extraction methodologies (e.g., using different solvents and mixtures of solvents), different derivatization methodologies, or different platforms for data acquisition (e.g., GC-FID) [57,58]. Despite a few common FA species, each microalga had a unique FA signature (Table 2), with microalgae from the same phylum showing similar profiles and clustering at higher levels, as observed in the principal component analysis (PCA) (Figure 2) and in the hierarchical clustering analysis (Figure S1). Omega-3 FAs have been identified with significantly elevated abundances in all microalgae, except *Spirulina* sp. The absence of ω-3 FA in *Spirulina* species has been extensively reported [51,59,60,61,62,63]. However, some studies described the presence of some of these PUFA, although in very low abundances [64,65,66,67,68]. These differences might be dependent on growth conditions, as ω-3 production is tightly dependent on them [3]. The microalga *S. obliquus* had the highest abundance of ω-3 PUFA (55.9 ± 4.5%), showing the highest abundance of α-linolenic acid (ALA, C18:3 ω-3) (35.0 ± 2.7%). This ω-3 is an essential FA of the greatest importance for humans and mammals, and can be metabolized into another longer ω-3 PUFA, such as eicosapentaenoic acid (EPA, C20:5 ω-3) and docosahexaenoic acid (DHA, C22:6 ω-3) [4]. These results are in line with what was previously reported for *S. obliquus*; however, some studies have reported high abundances of EPA in this microorganism, which we do not report [69,70,71,72]. Nonetheless, variations in cultivation might justify the absence of EPA, as salinity, nitrogen, and nutrient stress affect the composition and yield of FA [73,74,75,76]. Additionally, the way *S. obliquus* biomass is processed has been shown to affect the abundance of FA [69]. The high abundance of this essential FA in *S. obliquus* contributes to the valorization of this microalga as a source of this essential ω-3 FA as a food ingredient. However, the synthesis of EPA and DHA from ALA is limited in mammals, and they must also be absorbed through the diet [5]. These two ω-3 FAs were not found in *S. obliquus*. Nonetheless, EPA was found in significant high abundances in other microalgae, particularly in *N. oceanica*, which lacks ALA. In line with our results, previous studies reported a total absence of ALA and high abundances of EPA [77,78,79,80,81,82]. One study reported the presence of negligible amounts of ALA; however, the authors were evaluating the effects of the use of phytohormones and nitrogen depletion on biomass and lipid production, which might justify the presence of negligible amounts of this FA in *N. oceanica* [83]. Regarding these FAs, an interesting correlation was observed between the abundances of ALA and EPA. Namely, when ALA was the most abundant FA, EPA was either absent (*S. obliquus*) or in lower amounts (*C. amblystomatis* and *T. chui*), suggesting a lower synthesis of EPA from ALA. The reverse was also observed in microalgae with EPA as the most abundant FA having no ALA (*N. oceanica*) or negligible amounts (*P. tricornutum*), suggesting increased production of EPA. This relationship between ALA and EPA appears to be unrelated to saline and freshwater growth. *P. tricornutum* was the only microalga in this study containing DHA, but with negligible amounts (0.6 ± 0.1%). EPA and DHA FA are important for good neurological development, as well as a regulated inflammatory response, as they are precursors of anti-inflammatory eicosanoids [9,10]. Other authors also observed small abundances of DHA in *P. tricornutum* when compared to ALA and EPA [84,85,86,87,88]. Few studies even reported an absence of DHA in this microalga [89,90,91].

Omega-3 FA are also very important for the prevention of atherosclerosis and cardiovascular disease [92]. The use of cardiovascular disease risk predictors, such as the atherogenic index (AI) and the thrombogenic index (TI), facilitates evaluation of the nutritional quality of lipids since the AI and TI measure the probability of reducing the risk of atherogenic plaques and blood clots, respectively [93]. These indices have already been used to assess these benefits in seaweeds, fish, and even microalgae [67,94,95,96,97]. Among the microalgae studied, *S. obliquus* showed both the lowest AI (0.2 ± 0.1) and TI (0.1 ± 0.0) values. The index values reported in this study were similar to those reported for fish and other seafood [98,99]. Compared to the indices reported for other microalgae, *S. obliquus* showed lower AI and TI than *Spirulina platensis*, *Nannochloropsis gaditana*, *Nannochloropsis oculata*, and *Porphyridium cruentum* (values among 0.49–1.70 and 0.22–3.82, respectively) [67].

Antioxidant compounds are in great demand as food ingredients to prevent damage caused by oxidative stress and, at the same time, the development of various diseases, including NCDs [100]. They are also of interest as natural preservatives to prevent oxidation and spoilage of food. Seaweed extracts have already shown potential to prevent food spoilage [101,102]. Microalgae are natural sources of antioxidant agents because they produce phenolic compounds [103], described as powerful antioxidants [104], and antioxidant lipids [105], in particular PUFA [49], which scavenge oxidative radicals and prevent damage caused by oxidation [106]. Most studies have focused on the antioxidant potential of phenolic compounds from microalgae; however, individual studies have highlighted the antioxidant activity of their lipids [29,30,31,32,33]. The ABTS^●+^ and DPPH^●^ radical scavenging assays were used to assess this biological potential of lipid extracts from seven different microalgae. For the evaluation of the antioxidant potential, it is useful to use distinct methods as distinct compounds and different mechanisms may underly the antioxidant potential [107,108]. Among the different methods for antioxidant activity screening, the DPPH and ABTS assays are the most used ones due to their methodological simplicity and stability of the radicals. The ABTS assay evaluates the capacity of the extracts to reduce the ABTS^●+^ radical, which is generated previously through the oxidation of the ABTS, while the DPPH assay evaluates the capacity of the extracts to reduce the DPPH^●^ radical, which is a stabilized radical by itself [108,109]. Both assays were used to evaluate the antioxidant properties of the microalgae lipid extracts, with *T. chui* showing the highest IC20 in the DPPH assay. The obtained results suggest that each microalga has antioxidant potential but at different levels. *S. obliquus* showed the highest antioxidant potential of all the microalgae studied, with an IC50 for the ABTS^●+^ scavenging assay of 29.4 ± 1.2 μg/mL (TE of 637.5 ± 27.4), and with a DPPH^●^ IC20 value of 89.1 ± 6.6 μg/mL^−1^ (TE of 114.5 ± 8.9 µmol.g^−1^). A previous study reported the DPPH^●^ radical scavenging activity for methanol extracts of different microalgae, including *S. obliquus*, which promoted 20% inhibition at around 200 μg/mL^−1^, showing lower antioxidant power compared to dichloromethane:methanol extracts [49]. Our extracts also showed lower IC20 compared to seven other microalgae used in the same study. Another study using ultrasound-assisted ethanol extracts of *S. obliquus* reached IC50 of DPPH^●^ radical at 12.75 ± 0.31 µg.mL^−1^, showing higher antioxidant power compared to the extracts used in our study [110]. This microalga currently has no food safety approval [23]; however, the high content of ω-3 FA, favorable nutritional indicators, and the potent ability to scavenge the ABTS^●+^ radical indicate a promising food ingredient with health benefits, and a source of antioxidants to prevent spoilage of food. The highest IC50 for the ABTS^●+^ radical scavenging assay was attributed to extracts of *N. oceanica* (101.9 ± 1.7 µg.mL^−1^), with poorer performance than the remaining six microalgae. This could be associated with the lower abundance of PUFA in this microalga, when compared with the others in this study (Table 2), since the antioxidant activity in lipid extracts has been attributed to PUFA [49]. In fact, the microalgae with the best antioxidant activities (Figure 3) were the ones with a higher abundance of PUFA (*C. vulgaris*, *C. amblystomatis*, *S. obliquus*, and *P. tricornutum*). This was confirmed when calculating the correlation coefficient between PUFA and antioxidant activity (Figure S2), where PUFA were highly correlated with the TE obtained in the DPPH and ABTS assays, but TE was not correlated with the amount of MUFA. This can be justified since PUFAs are more prone to oxidation, as they are the first targets of free radicals [111,112,113]. In addition, despite the nutritional importance of the balance between omega-3 and omega-6 FAs, the relation with antioxidant activity through inhibition of free radicals appears to depend on the amount of PUFA but does not depend on the amount of omega 3 or omega 6 FAs. In the literature, few published works have reported a similar trend in the susceptibility to oxidation of both omega-3 and omega-6 FAs, while others suggested a dissimilar behavior [106,111,112]. In fact, *N. oceanica* have the high content in EPA (Table 1) but showed the lowest antioxidant capabilities in both ABTS and DPPH assays, when compared to the other microalgae (Figure 3). Nonetheless, *N. oceanica* might have antioxidant potential using other mechanisms. For instance, it was reported to enhance the expression of the antioxidant enzymes glutathione s-transferase, glutathione peroxidase, and glutathione reductase in Cornish Giant cockerels [113]. On the other hand, the lowest IC20 value for the DPPH^●^ assay was found for *C. vulgaris* of 50.5 ± 12.3 µg.mL^−1^ (TE 191.8 ± 40.0 µmol.g^−1^). Compared to hexane and hexane:chloroform extracts from another study, these extracts did not fare so well [114]. This microalga has current applications in the food industry, being used as a supplement or directly as food. The antioxidant potential described here is beneficial for its valorization as a new natural source of antioxidant agents, namely antioxidant lipids. In our study, the extracts with the highest IC20 were those of *T.chui* (225.7 ± 6.9 µg.mL^−1^). Previous studies using methanolic extracts of this microalga had a lower DPPH^●^ IC20 [49,115]. Another study reported 90.7% DPPH inhibition for ethanol extracts but at 10 mg/mL [116]. However, this microalga had the third lowest IC50 for the ABTS^●+^ radical, nevertheless suggesting that this microalga is able to prevent the oxidation of other radicals.

In summary, this study supports already existing studies in order to better understand the FA composition in microalgae. Different microalgae have different lipid composition, which can be valued and explored for different biotechnology applications and industries. With the exception of *Spirulina* sp., the microalgae in this study had high amounts of ω-3 FA, with *S. obliquus* showing the greatest abundance. This was favorable to the nutritional indices calculated for this microalga. In addition, *S. obliquus* had a better performance in the antioxidant assay, which strengthens the nutritional potential of this sustainable bio-factory for the implementation of food-based ingredients and preservatives. However, despite the extraction approach generating extracts rich in lipids, one cannot exclude the possible contribution of other compounds to the described antioxidant activity. Therefore, further studies are needed to explore and establish the relationship between microalgae lipids and biological activities. Nevertheless, the results described here reinforce the use of microalgae as a source of value-added lipids for the food, feed, and pharmaceutical industries.

## 4. Materials and Methods

### 4.1. Reagents

HPLC-grade methanol (MeOH), ethanol absolute, hexane, and dichloromethane (CH_2_Cl_2_) were purchased from Fisher Scientific Ltd. (Loughborough, UK). All other reagents were purchased from major commercial sources. Milli-Q water (Synergy, Millipore Corporation, Billerica, MA, USA) was used. Furthermore, 2,2-diphenyl-1-picrylhydrazy radical (DPPH^●^) was purchased from Aldrich (Milwaukee, WI), 2,20-Azino-bis (3-ethylbenzothiazoline-6-sulfonic acid) diammonium salt (ABTS^●+^) was obtained from Fluka (Buchs, Switzerland), and 6-hydroxy-2,5,7,8-tetramethylchromane-2-carboxylic acid (Trolox) was purchased from Sigma-Aldrich (St Louis, MO, USA). All the other reagents and chemicals used were of the highest grade of purity commercially available.

### 4.2. Microalgae Material

The spray-dried biomass of *Chlorella vulgaris*, *Chlorococcum amblystomatis*, *Scenedesmus obliquus*, *Tetraselmis chui*, *Phaeodactylum tricornutum*, *Spirulina* sp., and *Nannochloropsis oceanica* was supplied by Allmicroalgae, Natural Products S.A. located in Pataias, Portugal.

The specimens are internally deposited at Allmicroalgae’s culture collection. All species were autotrophically grown in Guillard’s F2 culture medium, the composition of which was adapted to the local water (REF below). *Phaeodactylum tricornutum*, *Tetraselmis chui*, and *Nannochloropsis oceanica* were further supplemented with magnesium mixture (Necton, Olhão, Portugal) and NaCl (Salexpor, Coimbra, Portugal) at 30 g/L salinity. *Spirulina* sp. was supplemented with 16.8 g/L sodium bicarbonate (Quimitécnica, Barreiro, Portugal). Then, 5-L flask reactors were cultivated from 7 to 15 days, under continuous 700 μmol photons.m^2^/s light exposition. Five 5-L flask reactors were used to inoculate one 0.1 m^3^ L outdoor flat panel (FP) reactor, which was later sequentially scaled until 1 m^3^ FPs was reached. Except in the case of *Spirulina* sp., which was collected directly from the FPs, four of the later reactors were used as inoculum of a 10 m^3^ tubular photobioreactor (PBR). The reactor was operated for the time necessary to reach the stationary phase, exposed to the environmental light and temperature conditions. Constant pH was maintained by pulse injections of CO_2_ and the temperature was kept under the limit by a sprinkler-like irrigation system. Table 3 describes the pH and temperature at which PBRs were operated. After the growing period, the biomass was industrially collected by centrifugation and further spray-drying.

### 4.3. Lipid Extraction Procedure

Lipids were extracted from 25 mg of lyophilized biomass of *Chlorella vulgaris*, *Chlorococcum amblystomatis*, *Scenedesmus obliquus*, *Tetraselmis chui*, *Phaeodactylum tricornutum*, *Spirulina* sp., and *Nannochloropsis oceanica*, based on Folch extraction [44,117], using dichloromethane instead of chloroform. The phase separation was achieved by centrifugation at 2000 rpm for 10 min, and the organic phase was collected. The aqueous phase was reextracted with 2 mL of dichloromethane, two more times. The combined organic phases were dried under a stream of nitrogen and weighted.

Each series of extracts was repeated five times and the total lipid content was determined by gravimetry.

### 4.4. Analysis of Fatty Acids by Gas Chromatography-Mass Spectrometry (GC-MS)

The fatty acid methyl esters (FAMEs) were prepared from total lipid extracts of *Chlorella vulgaris*, *Chlorococcum amblystomatis*, *Scenedesmus obliquus*, *Tetraselmis chui*, *Phaeodactylum tricornutum*, *Spirulina* sp., and *Nannochloropsis oceanica* by transmethylation reaction using a methanolic solution of potassium hydroxide (2.0 M) according to the methodology previously described [118]. A volume of 2.0 μL of a hexane solution containing FAMEs and 1.0 μg mL^−1^ of methyl nonadecanoate (Sigma, St. Louis, MO, USA) as internal standard was injected in a chromatography-mass spectrometry (GC–MS) (Agilent Technologies 8860 GC System, Santa Clara, CA, USA) equipped with a DB-FFAP column with the following specifications: 30 m long, 0.32 mm internal diameter, and 0.25 μm film thickness (J&W Scientific, Folsom, CA, USA). The GC equipment was connected to an Agilent 5977B Mass Selective Detector operating with electron impact ionization at 70 eV and a scanning range of *m/z* 50–550 (1 s cycle in a full scan mode). The following conditions were used: helium as carrier gas (constant flow 1.4 mL min^−1^), inlet temperature 220 °C, detector temperature 230 °C, and injection volume 2 μL (splitless). The oven temperature was programmed as follows: 58 °C for 2 min, 25 °C min^−1^ to 160 °C, 2 °C min^−1^ to 210 °C, and 30 °C min^−1^ to 225 °C (held for 20 min). The data acquisition software used was GCMS 5977B/Enhanced MassHunter. 

### 4.5. Data Analysis

The acquired data were analyzed using Agilent MassHunter Qualitative Analysis 10.0 software. The identification of FA was carried out by the retention time and comparison of the MS spectrum with the NIST chemical database library and confirmed with the literature reports [119]. Five independent replicates were injected. The atherogenic (AI), thrombogenic (TI) and hypocholesterolemic/hypercholesterolemic indexes (h/H) were calculated using the following formula (Equations (1)–(3)), as proposed by Ulbricht and Southgate [120]:

(1) $$\mathrm{AI}=\frac{[C12:0+4]\;\times \;[\left(C14:0\right)+C16:0]}{[\sum \mathrm{MUFA}+\sum \left(n\;-\;6\right)+\sum \left(n\;-\;3)\right]}$$

(2) $$\mathrm{TTI}=\frac{\left[C14:0+C16:0+C18:0\right]}{[0.5\times \sum \mathrm{MUFA}+0.5\times \sum \left(n\;-\;6\right)+3\times \sum \left(n\;-3\right)+(\frac{\sum \left(n\;-\;6\right)}{\sum \left(n\;-3\right)})]}$$

(3) $$\left(h/H\right)=\frac{\left[C18:1(\omega -9)+18:2(\omega -6)+18:3(\omega -3)+C20:4(\omega -6)+C20:5(\omega -3)\right]}{\left[C14:0+C16:0\right]}$$

### 4.6. Statistical Analysis

Multivariate and univariate analyses were performed using R version 4.0.2 [121] in Rstudio version 1.3.1093 [122]. Data were glog transformed using the Metaboanalyst software [123]. Principal component analysis (PCA) was conducted for exploratory data analysis, with the R built-in function. PCA and ellipses (level of 0.95) were created using R libraries FactoMineR [124] and factoextra [125]. Hierarchical clustering was generated by the Metaboanalyst [126]. The Kruskal–Wallis test followed by Dunn’s post-hoc comparisons were performed with the R built-in function. P-values were corrected for multiple testing using the BH Benjamini, Hochberg, and Yekutieli method (q values) [127]. A q-value < 0.05 was considered an indicator of statistical significance. All graphics and boxplots were created using the R package ggplot2 [128]. Other R packages used for data management and graphics included plyr [129], dplyr [130], and tidyr [131].

### 4.7. DPPH Radical Scavenging Assay

The antioxidant scavenging activity against the 2,2-diphenyl-1-picrylhydrazyl radical (DPPH^●^) was evaluated as described in previous studies [107,132], with some modifications. Firstly, 150 µL of an ethanolic dilution of the extracts (25, 125, 250, 500 µg mL^−1^) or 150 µL of the Trolox standard solution (5, 12.5, 25, 37.5 µmol L^−1^ in ethanol) were mixed in triplicate with 150 µL of a DPPH^●^ working solution in ethanol (absorbance ~0.9, 517 nm). The mixture was incubated for 120 min and the absorbance was measured at 517 nm every 5 min (Multiskan GO 1.00.38, Thermo Scientific, Hudson, NH, USA). A control was prepared by replacing the DPPH^●^ solution with ethanol. The antioxidant activity, expressed as a percentage of inhibition of the DPPH radical, was calculated using the following equation (Equation (4)):

(4) $$\mathrm{Inhibition}\%=\frac{\left({\mathrm{Abs}}_{\mathrm{DPPH}•}-{\mathrm{Abs}}_{\mathrm{Sample}-\mathrm{control}}\right)}{{\mathrm{Abs}}_{\mathrm{DPPH}•}}\times 100$$

The concentration of sample able to scavenge 20% of DPPH radical (IC20) after 120 min of reaction was calculated by linear regression plotting the concentration of lipid extract versus the percentage of the inhibition curve. The activity is expressed in Trolox Equivalents, which were calculated using Equation (5), where IC20 values are the concentration of sample or of Trolox that induces the reduction of the DPPH^•^ radical to 20%:

(5) $$TE=\frac{IC20\;Trolox\;\left(\mu \mathrm{mol}/g\right)}{IC20\;of\;samples\;\left(\mu g/\mathrm{mL}\right)}\times 1000$$

### 4.8. ABTS Cation Radical Scavenging Assay

The antioxidant scavenging activity against the 2,2′-azino-bis-3-ethylbenzothiazoline-6-sulfonic acid radical cation (ABTS^●+^) was evaluated using a method previously described [132,133] with some modifications. Firstly, 150 µL of an ethanolic dilution of the extracts (25, 125, 250, 500 µg mL^−1^) or 150 µL of the Trolox standard solution (5, 12.5, 25, 37.5 µmol L^−1^ in ethanol) were mixed in triplicate with 150 µL of an ABTS^●+^ working solution in ethanol (absorbance ≈0.9, 734 nm). The mixture was incubated for 120 min and the absorbance was measured at 734 nm every 5 min (Multiskan GO 1.00.38, Thermo Scientific, Hudson, NH, USA). A control was prepared by replacing the ABTS^●+^ solution with ethanol. The antioxidant activity, expressed as a percentage of inhibition of the ABTS radical, was calculated using Equations (1) and (2) (Abs_DPPH^●^ substituted by Abs_ABTS^●+^) and expressed in IC50 and Trolox Equivalents (Equation (2)).

## Figures and Tables

**Table 1:** Fatty acid (FA) profile of *Chlorella vulgaris*, *Chlorococcum amblystomatis*, *Scenedesmus obliquus*, *Tetraselmis chui*, *Phaeodactylum tricornutum*, *Spirulina* sp., and *Nannochloropsis oceanica* by GC-MS. FAs are expressed in relative abundance (%) and the values are the means of five analytical samples (n = 5) ± standard deviation (SD).

FA	Chlorophyta				Bacillariophyta	Cyanobacteria	Ochrophyta
	*Chlorella vulgaris*	*Chlorococcum amblystomatis*	*Scenedesmus obliquus*	*Tetraselmis chui*	*Phaeodactylum tricornutum*	*Spirulina* sp.	*Nannochloropsis oceanica*
**C12:0**							0.2 ± 0.0
**C14:0**	0.3 ± 0.0	1.4 ± 0.2	0.5 ± 0.1	0.5 ± 0.1	4.2 ± 0.3	0.1 ± 0.0	3.5 ± 0.1
**C15:0**	0.3 ± 0.0	0.2 ± 0.1	0.2 ± 0.0	0.2 ± 0.1	0.3 ± 0.0		0.2 ± 0.0
**C16:0**		21.5 ± 1.3	14.9 ± 2.2	24.2 ± 2.1	14.7 ± 0.9	38.6 ± 0.4	22.6 ± 2.0
**C16:1**			2.9 ± 0.2				
**C16:1 ∆^11^ (ω-5)**		0.5 ± 0.0	2.7 ± 0.2	0.4 ± 0.1	1.3 ± 0.1		0.7 ± 0.1
**C16:1 ∆^9^ (ω-7)**	2.4 ± 0.2	8.9 ± 0.5	1.3 ± 0.1	1.1 ± 0.1	16.4 ± 0.3	5.1 ± 0.1	21.6 ± 1.3
**C16:1 ∆^7^ (ω-9)**	0.9 ± 0.1		1.2 ± 0.1	0.7 ± 0.1	0.2 ± 0.0	1.6 ± 0.1	0.1 ± 0.0
**C16:2**					0.8 ± 0.0		
**C16:2 ∆^9,12^ (ω-4)**					5.1 ± 0.1	0.2 ± 0.0	
**C16:2 ∆^7,10^ (ω-6)**	6.7 ± 0.4	0.3 ± 0.0	0.3 ± 0.0	0.5 ± 0.1			0.1 ± 0.0
**C16:3 ∆^7,10,13^ (ω-3)**	14.2 ± 0.8	0.8 ± 0.0	0.7 ± 0.1	1.3 ± 0.2	0.2 ± 0.0		
**C16:3 ∆^6,9,12^ (ω-4)**					4.7 ± 0.1		
**C16:3 ∆^4,7,10^ (ω-6)**		0.7 ± 0.0	0.2 ± 0.0	0.4 ± 0.0			
**C16:4 ∆^6,9,12,15^ (ω-1)**					7.6 ± 0.2		
**C16:4 ∆^4,7,10,13^ (ω-3)**		12.7 ± 0.6	15.5 ± 1.4	14.5 ± 1.8	0.6 ± 0.0		
**C17:0**	0.7 ± 0.0	0.2 ± 0.0	0.3 ± 0.0	0.1 ± 0.0	0.1 ± 0.0	0.2 ± 0.0	0.1 ± 0.0
**C17:1**	0.5 ± 0.0	0.2 ± 0.0	0.1 ± 0.0			0.2 ± 0.0	0.4 ± 0.0
**C18:0**	6.5 ± 2.2	4.0 ± 2.1	9.7 ± 3.9	8.8 ± 6.3	3.7 ± 1.4	5.4 ± 2.1	6.3 ± 3.2
**C18:1 ∆^11^ (ω-7)**	2.3 ± 0.1	4.4 ± 0.2	1.8 ± 0.1	4.8 ± 0.6	2.6 ± 0.1	1.4 ± 0.1	0.5 ± 0.1
**C18:1 ∆^9^ (ω-9)**	4.1 ± 0.2	2.2 ± 0.1	4.3 ± 0.6	7.0 ± 0.9	1.3 ± 0.1	2.6 ± 0.1	4.1 ± 0.3
**C18:2 ∆^9,12^ (ω-6)**	17.6 ± 0.9	2.9 ± 0.2	3.5 ± 0.3	3.6 ± 0.5	3.1 ± 0.1	21.4 ± 0.8	3.2 ± 0.3
**C18:3 ∆^9,12,15^ (ω-3)**	26.3 ± 1.4	21.9 ± 0.9	35.0 ± 2.7	18.3 ± 2.4	1.8 ± 0.1		
**C18:3 ∆^6,9,12^ (ω-6)**		1.3 ± 0.1	0.4 ± 0.1	2.3 ± 0.3	0.2 ± 0.0	23.3 ± 0.8	0.2 ± 0.0
**C18:4 ∆^6,9,12,15^ (ω-3)**		4.0 ± 0.2	4.7 ± 0.4	6.3 ± 0.9	0.5 ± 0.0		
**C20:1 ∆^11^**				0.6 ± 0.1			
**C20:3 ∆^5,11,14^ (ω-6)**					0.3 ± 0.1		
**C20:4 ∆^5,11,14,17^ (ω-3)**					0.3 ± 0.0		
**C20:4 ∆^5,8,11,14^ (ω-6)**		1.5 ± 0.1		0.3 ± 0.1	1.1 ± 0.1		5.3 ± 0.6
**C20:5 ∆^5,8,11,14,17^ (ω-3)**		10.7 ± 0.6		4.2 ± 0.6	27.3 ± 1.5		30.8 ± 2.4
**C22:6 ∆^4,7,10,13,16,19^ (ω-3)**					0.6 ± 0.1		
**C24:0**					0.9 ± 0.1		

**Table 2:** Fatty acid (FA) indicators of *Chlorella vulgaris*, *Chlorococcum amblystomatis*, *Scenedesmus obliquus*, *Tetraselmis chui*, *Phaeodactylum tricornutum*, *Spirulina* sp., and *Nannochloropsis oceanica*. Values correspond to relative abundances (except for AI, TI, and h/H calculations) and are the means of five analytical samples (n = 5) ± standard deviation (SD). Matching letters (a–l) indicate statistically significant differences between microalga species, i.e., the same letter represents significant differences (*q* < 0.05, Kruskal–Wallis test followed by Dunn’s post-hoc comparisons).

Indicators	Chlorophyta				Bacillariophyta	Cyanobacteria	Ochrophyta
	*Chlorella vulgaris*	*Chlorococcum amblystomatis*	*Scenedesmus obliquus*	*Tetraselmis chui*	*Phaeodactylum tricornutum*	*Spirulina* sp.	*Nannochloropsis oceanica*
**∑ SFA**	24.9 ± 4.1	27.3 ± 3.3	25.5 ± 5.7	33.6 ± 8.4	23.8 ± 2.4	44.2 ± 1.8	33.0 ± 5.1
**∑ MUFA**	10.3 ± 0.6	16.1 ± 0.8	14.3 ± 1.1	14.5 ± 1.8	21.9 ± 0.5	10.9 ± 0.3	27.4 ± 1.8
**∑ PUFA**	64.6 ± 3.5	56.7 ± 2.6	60.1 ± 4.8	51.8 ± 6.6	54.3 ± 2.0	44.8 ± 1.5	39.7 ± 3.4
**∑ PUFA ω-6**	24.3 ± 1.4	6.7 ± 0.4	4.2 ± 0.4	7.3 ± 1.0	4.8 ± 0.3	44.6 ± 1.5	8.9 ± 0.9
**∑ PUFA ω-3**	40.5 ± 2.2	50.0 ± 2.2	55.9 ± 4.5	44.6 ± 5.7	31.3 ± 1.7	--	30.8 ± 2.4
**ω-6/ω-3 ratio**	0.6 ± 0.0	0.1 ± 0.0	0.1 ± 0.0	0.2 ± 0.0	0.2 ± 0.0	--	0.3 ± 0.0
**AI**	0.2 ± 0.0 ^a,b,c^	0.4 ± 0.0 ^d^	0.2 ± 0.1 ^e,f,g^	0.4 ± 0.1	0.5 ± 0.1 ^a,f^	0.7 ± 0.0 ^b,d,g^	0.6 ± 0.1 ^c,e^
**TI**	0.2 ± 0.0 ^b^	0.2 ± 0.0 ^h,i^	0.1 ± 0.0 ^e,g^	0.2 ± 0.1	0.2 ± 0.2	1.6 ± 0.1 ^b,g,h^	0.3 ± 0.1 ^e,i^
**(h/H)**	2.8 ± 0.4 ^b,j^	1.7 ± 0.2	2.9 ± 0.5 ^g,k^	1.4 ± 0.3 ^j,k^	1.8 ± 0.2 ^l^	0.6 ± 0.0 ^b,g,l^	1.7 ± 0.2

SFA, saturated fatty acids; MUFA, monounsaturated fatty acids; PUFA, polyunsaturated fatty acids; AI, Atherogenicity index; TI, Thrombogenicity index; (h/H), hypocholesterolemic/hypercholesterolemic ratio.

**Table 3:** Description of the limiting temperature and pH conditions operated in the tubular photobioreactors for microalgae cultivation.

Microalgae	Temperature (°C)	pH
*Chlorella vulgaris*	<25	7.5–8.5
*Chlorococcum amblystomatis*	<28	7.5–8.5
*Scenedesmus obliquus*	<25	7.5–8.5
*Tetraselmis chui*	<25	7.5–8.5
*Phaeodactylum tricornutum*	<23	7.5–8.5
*Nannochloropsis oceanica*	<25	7.5–8.5

## Data Availability

Raw datasets generated during this study are available from the corresponding authors upon reasonable request.

## Supplementary Materials

The following are available online at https://www.mdpi.com/article/10.3390/md19070357/s1 Table S1. Lipid content of *Chlorella vulgaris*, *Chlorococcum amblystomatis*, *Scenedesmus obliquus*, *Tetraselmis chui*, *Phaeodactylum tricornutum*, *Spirulina* sp. and *Nannochloropsis oceanica*. Kruskal–Wallis test followed by Dunn’s post-hoc comparisons). Table S2. Evaluation of the antioxidant activity for the extracts of different microalgae. Concentration of lipid extract (µg.mL^−1^) providing 50% inhibition of the ABTS^●+^ radical and 20% inhibition of the DPPH^●^ radical, and their respective Trolox equivalents (TE; µmol.g^−1^). The values are displayed as the mean (n = 3) ± standard deviation. Matching letters (a–f) indicate statistically significant differences between microalgae species, i.e., the same letter repre-sents significant differences (*q* < 0.05, Kruskal–Wallis test followed by Dunn’s post-hoc comparisons). Figure S1. (A) Hierarchical cluster analysis using relative abundance after Glog normalization of all fat-ty acids identified in *Chlorella vulgaris*, *Chlorococcum amblystomatis*, *Scenedesmus obliquus*, *Tetraselmis chui*, *Phaeodactylum tricornutum*. *Spirulina* sp. and *Nannochloropsis oceanica*. The green, blue, yellow, and orange boxes show microalgae from the phylum Chlorophyta, Ochrophyta, Bacillariophyta and Cyanobacteria, respectively. Abbreviations: C, *Chlorococcum amblystomatis*; C.v, *Chlorella vulgaris*; SC, *Scenedesmus obliquus*; T.C, *Tetraselmis chui*; Phae, *Phaeodactylum tricornutum*; SP, *Spirulina* sp.; and N.O, *Nannochloropsis oceanica*., Figure S2. Correlation coefficients analysis using the sum of relative abundances from saturated fatty acids (SFA), monounsaturated FA (MUFA), polyunsaturated FA (PUFA), omega-3 PUFA and omega-6 PUFA, and antioxidant activity. (A) Correlation coefficients with ABTS assay. (B) Correlation coefficients with DPPH assay.
