# Supplementary material for: Microalgae as Sustainable Bio-Factories of Healthy Lipids: Evaluating Fatty Acid Content and Antioxidant Activity
Source: Mar Drugs. 2021 Jun 23;19(7):357. doi: 10.3390/md19070357 (PMC8307217; doi:10.3390/md19070357)
Supplement: Supplementary file 1 [file marinedrugs-19-00357-s001.zip › marinedrugs-1241088-supplementary conversion.pdf]

Microalgae as sustainable bio-factories of healthy lipids: evaluating fatty acid content and antioxidant activity

Tiago A. Conde[1,2,3†], Bruna F. Neves[1,2†], Daniela Couto[1,2], Tânia Melo[1,2], Bruno Neves[3], Margarida Costa[4], Joana Silva[4], Pedro Domingues[1] and M. Rosário Domingues[1,2]

[1]Mass Spectrometry Centre, LAQV-REQUIMTE, Department of Chemistry, University of Aveiro, Santiago University Campus, 3810-193 Aveiro, Portugal

[2]CESAM - Centre for Environmental and Marine Studies, Department of Chemistry, University of Aveiro, Santiago University Campus, 3810-193 Aveiro, Portugal

[3]Department of Medical Sciences and Institute of Biomedicine – iBiMED, University of Aveiro, 3810-193 Aveiro, Portugal

[4]Allmicroalgae Natural Products S.A., R&D Department, Rua 25 de Abril 19, 2445-287 Pataias, Portugal

†both authors contributed equally for the manuscript

Corresponding author: mrd@ua.pt

Table S1. Lipid content of *Chlorella vulgaris, Chlorococcum amblystomatis, Scenedesmus obliquus, Tetraselmis chui, Phaeodactylum tricornutum, Spirulina* sp. and *Nannochloropsis oceanica.* Kruskal–Wallis test followed by Dunn's post-hoc comparisons).

**Microalgae**	***Chlorophyta***				***Bacillariophyta***	***Cyanobacteria***	***Ochrophyta***
	Chlorella vulgaris	***Chlorococcum amblystomatis***	***Scenedesmus obliquus***	***Tetraselmis chui***	***Phaeodactylum tricornutum***	***Spirulina* sp.**	***Nannochloropsis oceanica***
**Lipid content (%)**	8.8 ±0.7 [a,b]	16.6 ±0.9 [a,c]	11.1 ±1.1	6.5 ±0.4 [c,d,e]	12.6 ±1.2 [e]	10.7 ±1.0 [f]	**20.9 ±3.4 [b,d,f]**

Table S2. – Evaluation of the antioxidant activity for the extracts of different microalgae. Concentration of lipid extract (μg.mL^{-1}) providing 50% inhibition of the ABTS$^{•+}$ radical and 20% inhibition of the DPPH• radical, and their respective Trolox equivalents (TE; μmol.g^{-1}). The values are displayed as the mean (n = 3) ± standard deviation. Matching letters (a-f) indicate statistically significant differences between microalgae species, i.e. the same letter represents significant differences (q < 0.05, Kruskal–Wallis test followed by Dunn's post-hoc comparisons).

		Chlor.ophyta				***Bacillariophyta***	***Cyanobacteria***	***Ochrophyta***
		C. vulgaris	***C. amblystomatis***	***S. obliquus***	***T. chuui***	***P. tricornutum***	***Spirulina* sp.**	***N. oceanica***
**ABTS$^{•+}$**	IC50 (μg.mL^{-1})	51.1 ±3.7	52.6 ±4.6	29.4 ±1.2 [a,b]	40.9 ±4.7	57.3 ±4.5[b]	38.7 ±1.6[c]	101.9 ±1.7[a,c]
	TE (μmol.g^{-1})	368.4 ±27.0	358.8 ±32.4	637.5 ±27.4[a]	432.9 ±51.3	306.6 ±23.5	452.2 ±9.6	184.0 ±3.2[a]
**DPPH^{+}**	IC20 (μg.mL^{-1})	50.5 ±12.3[d,e]	58.4 ±10.7[f]	89.1 ±6.6	225.7 ±6.9[d,f]	75.4 ±4.6	96.9 ±9.7	175.6 ±8.7[e]
	TE (μmol.g^{-1})	191.8 ±40.0[d,e]	143.0 ±25.1[f]	114.5 ±8.9	45.0 ±1.4[d,f]	122.3 ±7.2	84.3 ±9.1	52.5 ±2.7 [e]

A. Hierarchical clustering

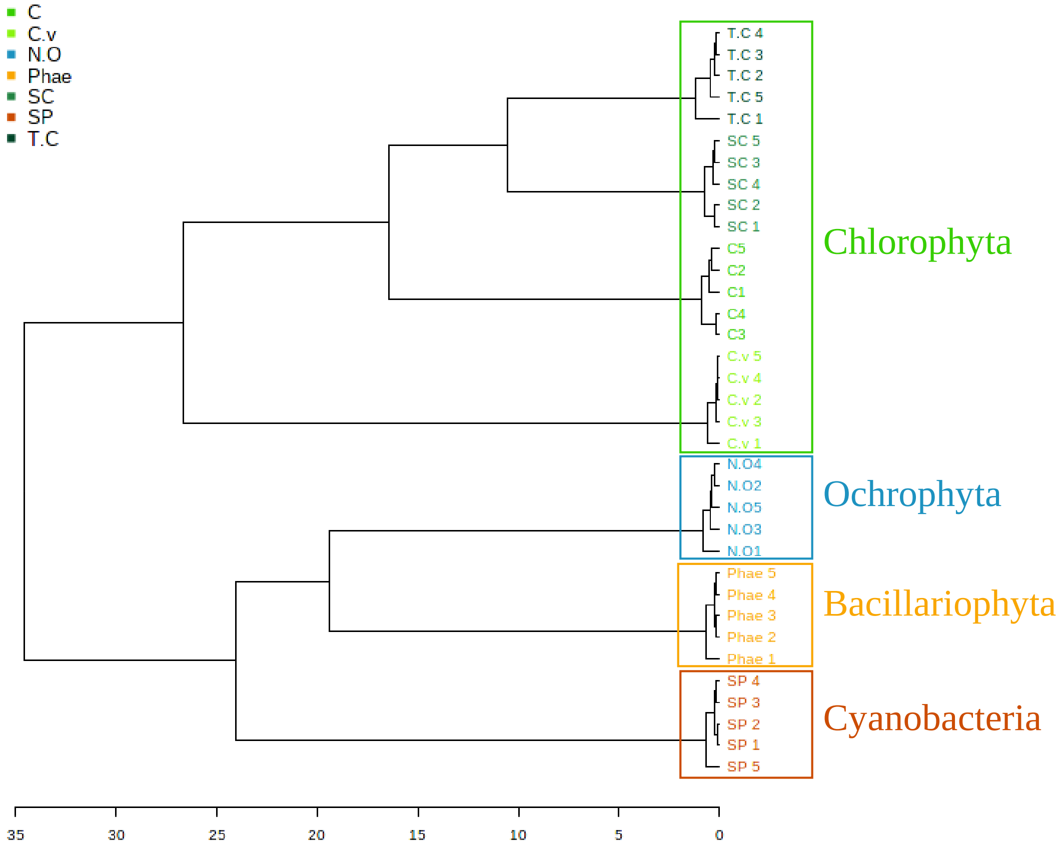

Figure S1. (**A**) Hierarchical cluster analysis using relative abundance after Glog normalization of all fatty acids identified in *Chlorella vulgaris, Chlorococcum amblystomatis, Scenedesmus obliquus, Tetraselmis chui, Phaeodactylum tricornutum., Spirulina* sp. and *Nannochloropsis oceanica*. The green, blue, yellow, and orange boxes show microalgae from the phylum Chlorophyta, Ochrophyta, Bacillariophyta and Cyanobacteria, respectively. Abbreviations: C, *Chlorococcum amblystomatis;* C.v, *Chlorella vulgaris;* SC, *Scenedesmus obliquus;* T.C, *Tetraselmis chui;* Phae, *Phaeodactylum tricornutum;* SP, *Spirulina* sp.; and N.O, *Nannochloropsis oceanica.*

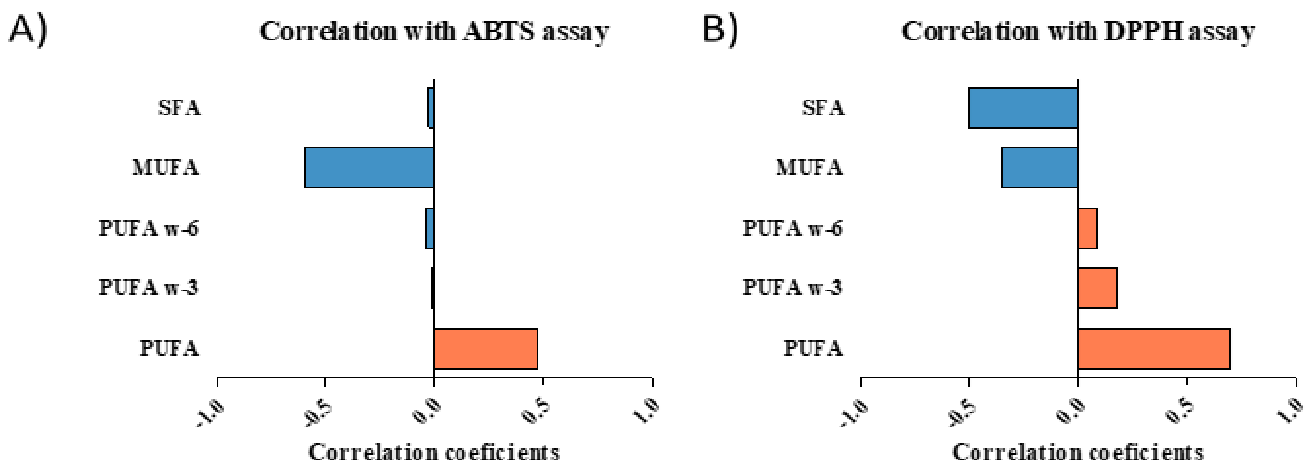

Figure S2. Correlation coefficients analysis using the sum of relative abundances from saturated fatty acids (SFA), mono-unsaturated FA (MUFA), polyunsaturated FA (PUFA), omega-3 PUFA and omega-6 PUFA, and antioxidant activity. (**A**) Correlation coefficients with ABTS assay. (**B**) Correlation coefficients with DPPH assay.
